# Reproducibility of Circulating MicroRNAs in Stored Plasma Samples

**DOI:** 10.1371/journal.pone.0136665

**Published:** 2015-08-27

**Authors:** Monica L. Bertoia, Kimberly A. Bertrand, Sherilyn J. Sawyer, Eric B. Rimm, Kenneth J. Mukamal

**Affiliations:** 1 Department of Nutrition, Harvard T. H. Chan School of Public Health, Boston, Massachusetts, United States of America; 2 Channing Division of Network Medicine, Department of Medicine, Brigham and Women's Hospital and Harvard Medical School, Boston, Massachusetts, United States of America; 3 Department of Epidemiology, Harvard T. H. Chan School of Public Health, Boston, Massachusetts, United States of America; 4 Department of Medicine, Beth Israel Deaconess Medical Center, Boston, Massachusetts, United States of America; National University of Singapore, SINGAPORE

## Abstract

**Background:**

Most studies of microRNA (miRNA) and disease have examined tissue-specific expression in limited numbers of samples. The presence of circulating miRNAs in plasma samples provides the opportunity to examine prospective associations between miRNA expression and disease in initially healthy individuals. However, little data exist on the reproducibility of miRNAs in stored plasma.

**Methods:**

We used Real-Time PCR to measure 61 pre-selected microRNA candidates in stored plasma. Coefficients of variation (CVs) were used to assess inter-assay reliability (n = 15) and within-person stability over one year (n = 80). Intraclass correlation coefficients (ICCs) and polychoric correlation coefficients were used to assess within-person stability and delayed processing reproducibility (whole blood stored at 4°C for 0, 24 and 48 hours; n = 12 samples).

**Results:**

Of 61 selected miRNAs, 23 were detected in at least 50% of samples and had average CVs below 20% for inter-assay reproducibility and 31 for delayed processing reproducibility. Ten miRNAs were detected in at least 50% of samples, had average CVs below 20% and had ICCs above 0.4 for within-person stability over 1–2 years, six of which satisfied criteria for both interassay reproducibility and short-term within-person stability (miR-17-5p, -191-5p, -26a-5p, -27b-3p, -320a, and -375) and two all three types of reproducibility (miR-27b-3p and -26a-5p). However, many miRNAs with acceptable average CVs had high maximum CVs, most had low expression levels, and several had low ICCs with delayed processing.

**Conclusions:**

About a tenth of miRNAs plausibly related to chronic disease were reliably detected in stored samples of healthy adults.

## Introduction

Micro RNAs (miRNAs) have recently emerged as key post-transcriptional regulators of gene expression. MiRNAs are ~22bp single-stranded RNA segments that silence gene expression by binding to complementary messenger RNA (mRNA). This binding represses translation and speeds mRNA degradation [1]. Unlike mRNA, miRNAs are remarkably stable and many are detectable in human plasma, several of which have been associated with cardiovascular disease (CVD) [2] and cancer [3].

Although the number of promising studies examining miRNAs related to CVD and cancer has grown substantially in recent years, most studies in humans have been cross-sectional and generally limited to 300 or fewer subjects [2]. Large, prospective cohorts with long-term stored plasma samples provide a unique opportunity to economically and efficiently study the prospective relationship between circulating miRNA expression and chronic disease. Analyses of miRNA expression in relation to chronic disease such as CVD and cancer will advance our knowledge of the biology of miRNA and may elucidate miRNAs as valuable biomarkers for disease risk prediction. To date, the reliability and reproducibility of miRNA measurement in stored plasma samples has yet to be confirmed.

To evaluate the feasibility of miRNA measurement in large-scale clinical studies, we constructed experiments to examine the inter-assay reproducibility of miRNA in stored plasma, measured by high-throughput quantitative Real-Time PCR (qRT-PCR). Many studies that span a large geographical region require specimen processing delays while samples are shipped to a central laboratory [4,5,6]. To examine the impact of delayed processing time on miRNA analyses, we examined reproducibility in sets of samples with a controlled delay in processing. Finally, in order to determine the validity of miRNA expression as a predictor of long-term associations with long latency diseases like cardiovascular disease and cancer [7,8], we examined the stability / variability of miRNA levels within individuals over one year.

## Materials and Methods

### Study Population

The Health Professionals Follow-up Study (HPFS) began in 1986 with the recruitment of 51,529 male health professionals age 40 to 75 years, 18,225 of which donated blood samples between 1993 and 1995 [9]. A subset of HPFS participants participated in a lifestyle validation study and donated an additional blood sample between 2012 and 2013. HPFS participants donated blood in three 10-mL ethylenediaminetetraacetic acid (EDTA) tubes that were shipped to a central laboratory overnight with an icepack in a Styrofoam container [10]. On arrival, blood samples were centrifuged, separated into aliquots of plasma, red blood cells, and buffy coat and stored in the vapor phase of liquid nitrogen, below -150°C.

### Reproducibility and Delay to Processing Experiments

To assess inter-assay reproducibility, we used two aliquots of EDTA plasma from each of 15 HPFS participants (Table 1). These plasma samples have been stored below -150°C for 1–2 years. To assess reproducibility with processing delays, we used three aliquots of EDTA plasma from 12 non-HPFS male volunteers aged 19–59 years that were (1) processed immediately, (2) stored at 4°C for 24 hours before processing and freezing, and (3) stored at 4°C for 48 hours before processing and freezing. The delayed processing plasma samples have been stored in the vapor phase of liquid nitrogen for 4–5 years.

**Table 1:** Description of inter-assay reproducibility, reproducibility with processing delays, and short-term within-person stability experiments.

**Experiment**	Inter-assay reproducibility	Reproducibility with processing delays	Short-term within-person reproducibility
**Participants**	HPFS	Non-HPFS	HPFS
**N**	15	12	80 (40 healthy and 40 less healthy*)
**Sample**	EDTA plasma	EDTA plasma	EDTA plasma
**Number of aliquots**	2	3	2
**Timing**		(1) Processed immediately	Donated mean 9.4
	Split sample	(2) Stored 24 h at 4°C	(range 6.5 to 12.9)
		(3) Stored 48 h at 4°C	months apart
**Storage conditions**	Liquid nitrogen (below -150°C)	Liquid nitrogen (below -150°C)	Liquid nitrogen (below -150°C)
	for 1–2 years	for 4–5 years	for 13–14 years

HPFS = Health Professionals Follow-up Study

* As indicated by smoking status, age (≤ or > 65 years), BMI (normal weight/overweight), and/or hypertensive state.

To assess short-term reproducibility within participants, we used two EDTA plasma samples from 80 HPFS participants donated at two distinct times with a mean of 9.4 (range 6.5 to 12.9) months apart. These plasma samples were mailed overnight to a central laboratory in a styrofoam container with an icepack (at approximately 4°C) and stored in the vapor phase of liquid nitrogen for 13–14 years. We included samples from 40 healthy and 40 less healthy participants (as indicated by smoking status, age (≤ or > 65 years), BMI (normal weight or overweight), and/or hypertensive state), hypothesizing that the larger degree of inter-individual variability in health status might lead to greater inter-individual variance and therefore higher intra-class correlation.

### MicroRNA Measurement

We selected 61 candidate miRNAs that have been reported to be detectable in circulation and have been cross-sectionally associated with cardiovascular disease [11,12,13,14,15,16,17,18]. These 61 candidate miRNAs (listed in Table 2) are included on commercial platforms, such as those by Exiqon (Woburn, MA) and Qiagen/SABiosciences (Valencia, CA).

**Table 2:** Mean expression levels (Ct), imputing 28 for samples with undetectable levels.

	Inter-assay		Reproducibility with processing		Short-term within	
	reproducibility		delays		person reproducibility	
	(n = 15 x 2)		(n = 12 x 3)		(n = 80 x 2)	
	N (%) detectable levels	Mean (SD)	N (%) detectable levels	Mean (SD)	N (%) detectable levels	Mean (SD)
hsa-let-7d-3p	0 (0%)	28.0 (0.0)	0 (0%)	28.0 (0.0)	2 (1%)	28.0 (0.4)
hsa-let-7d-5p	12 (40%)	27.1 (1.4)	32 (89%)	22.7 (2.5)	120 (75%)	24.8 (2.5)
hsa-let-7e-5p	0 (0%)	28.0 (0.0)	4 (11%)	27.5 (1.5)	22 (14%)	27.4 (1.7)
hsa-miR-1	0 (0%)	28.0 (0.0)	0 (0%)	28.0 (0.0)	0 (0%)	28.0 (0.0)
hsa-miR-106b-5p	28 (93%)	17.0 (3.2)	36 (100%)	12.9 (1.3)	160 (100%)	13.3 (1.9)
hsa-miR-10a-5p	0 (0%)	28.0 (0.0)	0 (0%)	28.0 (0.0)	24 (15%)	27.6 (1.0)
hsa-miR-122-5p	4 (13%)	27.5 (1.7)	13 (36%)	25.7 (3.5)	82 (51%)	25.5 (3.0)
hsa-miR-126-3p	28 (93%)	17.0 (3.3)	36 (100%)	12.6 (1.7)	160 (100%)	13.9 (1.8)
hsa-miR-126-5p	3 (10%)	27.4 (2.0)	28 (78%)	20.3 (5.4)	158 (99%)	17.7 (3.7)
hsa-miR-130a-3p	27 (90%)	20.2 (3.2)	36 (100%)	15.5 (2.1)	159 (99%)	15.6 (1.9)
hsa-miR-130b-3p	2 (7%)	27.8 (0.9)	21 (58%)	25.0 (3.0)	117 (73%)	24.5 (2.8)
hsa-miR-130b-5p	0 (0%)	28.0 (0.0)	0 (0%)	28.0 (0.0)	2 (1%)	28.0 (0.3)
hsa-miR-133a	8 (27%)	27.2 (1.7)	27 (75%)	23.4 (3.2)	95 (59%)	24.6 (3.5)
hsa-miR-133b	0 (0%)	28.0 (0.0)	0 (0%)	28.0 (0.0)	7 (4%)	27.8 (1.2)
hsa-miR-140-3p	10 (33%)	27.3 (1.0)	29 (81%)	25.1 (1.8)	146 (91%)	23.6 (2.4)
hsa-miR-142-5p	0 (0%)	28.0 (0.0)	11 (31%)	27.3 (1.2)	44 (28%)	27.2 (1.5)
hsa-miR-145-3p	0 (0%)	28.0 (0.0)	0 (0%)	28.0 (0.0)	122 (76%)	24.9 (2.5)
hsa-miR-145-5p	3 (10%)	27.8 (0.7)	33 (92%)	23.3 (2.4)	3 (2%)	28.0 (0.2)
hsa-miR-151a-3p	0 (0%)	28.0 (0.0)	1 (3%)	27.9 (0.3)	125 (78%)	24.5 (2.6)
hsa-miR-151a-5p	0 (0%)	28.0 (0.0)	1 (3%)	27.5 (2.8)	55 (34%)	26.8 (2.6)
hsa-miR-155-5p	2 (7%)	27.0 (3.9)	0 (0%)	28.0 (0.0)	0 (0%)	28.0 (0.0)
hsa-miR-17-5p	20 (67%)	23.4 (3.9)	36 (100%)	16.6 (3.0)	159 (99%)	16.2 (3.3)
hsa-miR-185-5p	6 (20%)	27.2 (1.7)	33 (92%)	22.5 (2.2)	155 (97%)	21.5 (2.2)
hsa-miR-191-3p	0 (0%)	28.0 (0.0)	1 (3%)	27.9 (0.5)	8 (5%)	27.8 (1.0)
hsa-miR-191-5p	28 (93%)	16.0 (3.7)	36 (100%)	11.6 (1.8)	160 (100%)	13.6 (2.4)
hsa-miR-193b-3p	0 (0%)	28.0 (0.0)	1 (3%)	27.8 (1.1)	76 (48%)	26.2 (2.3)
hsa-miR-199a-3p	26 (87%)	23.8 (2.4)	35 (97%)	18.8 (2.6)	160 (100%)	19.3 (1.9)
hsa-miR-199a-5p	0 (0%)	28.0 (0.0)	4 (11%)	27.6 (1.2)	3 (2%)	28.0 (0.3)
hsa-miR-20b-5p	15 (50%)	26.5 (1.7)	32 (89%)	23.7 (2.4)	160 (100%)	19.7 (2.2)
hsa-miR-21-5p	24 (80%)	24.5 (2.3)	34 (94%)	19.1 (3.1)	158 (99%)	17.7 (2.9)
hsa-miR-210	0 (0%)	28.0 (0.0)	5 (14%)	27.6 (1.2)	135 (84%)	22.7 (3.5)
hsa-miR-22-3p	0 (0%)	28.0 (0.0)	1 (3%)	28.0 (0.2)	39 (24%)	27.2 (1.7)
hsa-miR-22-5p	3 (10%)	25.6 (7.3)	0 (0%)	28.0 (0.0)	127 (79%)	24.3 (2.9)
hsa-miR-221-3p	28 (93%)	16.8 (3.4)	36 (100%)	12.5 (1.8)	159 (99%)	13.4 (1.9)
hsa-miR-222-3p	12 (40%)	26.9 (1.6)	33 (92%)	21.8 (2.7)	156 (98%)	21.9 (2.5)
hsa-miR-223-5p	1 (3%)	27.8 (0.9)	8 (22%)	27.4 (1.4)	153 (96%)	22.2 (2.8)
hsa-miR-23b-3p	0 (0%)	28.0 (0.0)	0 (0%)	28.0 (0.0)	0 (0%)	28.0 (0.0)
hsa-miR-23b-5p	1 (3%)	27.2 (4.2)	0 (0%)	28.0 (0.0)	0 (0%)	28.0 (0.0)
hsa-miR-25-3p	0 (0%)	28.0 (0.0)	0 (0%)	28.0 (0.0)	160 (100%)	8.2 (1.7)
hsa-miR-26a-5p	28 (93%)	19.7 (3.6)	36 (100%)	13.2 (2.5)	158 (99%)	18.5 (3.2)
hsa-miR-26b-3p	1 (3%)	27.9 (0.4)	0 (0%)	28.0 (0.0)	26 (16%)	27.5 (1.2)
hsa-miR-26b-5p	18 (60%)	25.7 (2.4)	35 (97%)	18.4 (2.9)	155 (97%)	19.9 (3.3)
hsa-miR-27a-3p	28 (93%)	18.1 (3.3)	36 (100%)	13.4 (1.8)	160 (100%)	14.4 (1.9)
hsa-miR-27b-3p	30 (100%)	13.5 (1.5)	36 (100%)	13.0 (1.1)	160 (100%)	16.5 (2.4)
hsa-miR-29a-3p	1 (3%)	27.9 (0.5)	24 (67%)	25.2 (2.4)	157 (98%)	22.3 (2.1)
hsa-miR-29b-3p	30 (100%)	20.0 (1.1)	36 (100%)	19.1 (1.0)	160 (100%)	17.7 (2.2)
hsa-miR-29c-3p	30 (100%)	14.1 (3.5)	36 (100%)	13.1 (2.7)	160 (100%)	14.2 (2.9)
hsa-miR-29c-5p	0 (0%)	28.0 (0.0)	0 (0%)	28.0 (0.0)	5 (3%)	27.9 (0.5)
hsa-miR-30b-5p	28 (93%)	16.9 (3.3)	36 (100%)	13.2 (1.5)	160 (100%)	12.7 (2.1)
hsa-miR-30c-5p	30 (100%)	11.5 (1.5)	36 (100%)	10.2 (0.8)	160 (100%)	11.1 (1.9)
hsa-miR-30e-3p	30 (100%)	16.8 (2.2)	35 (97%)	16.3 (2.8)	158 (99%)	12.9 (3.2)
hsa-miR-320a	26 (87%)	21.4 (3.3)	36 (100%)	17.4 (2.3)	160 (100%)	14.8 (2.0)
hsa-miR-320b	30 (100%)	6.7 (1.3)	35 (97%)	7.3 (3.6)	158 (99%)	7.9 (3.0)
hsa-miR-361-5p	0 (0%)	28.0 (0.0)	6 (17%)	27.5 (1.2)	109 (68%)	25.0 (2.7)
hsa-miR-375	16 (53%)	24.7 (3.4)	34 (94%)	19.9 (2.6)	159 (99%)	19.3 (1.7)
hsa-miR-409-3p	21 (70%)	22.3 (4.5)	34 (94%)	17.0 (3.9)	158 (99%)	14.2 (2.3)
hsa-miR-424-3p	1 (3%)	27.2 (4.3)	0 (0%)	28.0 (0.0)	4 (3%)	27.9 (0.6)
hsa-miR-424-5p	1 (3%)	27.2 (4.2)	0 (0%)	28.0 (0.0)	0 (0%)	28.0 (0.0)
hsa-miR-92a-3p	28 (93%)	11.0 (4.7)	36 (100%)	8.3 (0.9)	160 (100%)	6.6 (1.3)
hsa-miR-93-3p	0 (0%)	28.0 (0.0)	0 (0%)	28.0 (0.0)	131 (82%)	24.3 (2.5)
hsa-miR-93-5p	27 (90%)	21.3 (2.8)	36 (100%)	17.9 (1.8)	160 (100%)	15.6 (2.0)

MiRNAs were measured in blinded samples by the High Throughput Gene Expression Core Laboratory at the University of Massachusetts Medical School (Worcester, MA). All reproducibility and delayed processing samples were measured on one plate in August 2013, and all within-person stability samples were measured on a second plate in January 2014. After thawing, plasma samples were inverted 10 times rather than centrifuged to prevent possible lysing of white blood cells that may be present in plasma and contain their own miRNAs.

Total RNA was isolated and purified using the miRCURY RNA Isolation Kit-Biofluids (Exiqon, Woburn, MA) and manufacturer’s protocol. Isolated RNAs were reverse-transcribed into cDNA using the TaqMan MicroRNA Reverse Transcription Kit (Applied Biosystems, Foster City, CA) and manufacturer’s protocol. Primers were used for 61 candidate miRNAs that have been cross-sectionally associated with CVD in published studies. All cDNA samples were stored at -80°C until Real-Time PCR analysis.

After reverse transcription, a preamplification reaction was performed using the TaqMan PreAmp Master Mix 2X (Applied Biosystems, Foster City, CA) and the MegaPlex Human PreAmp Primer Pools Set v3.0 (Applied Biosystems, Foster City, CA) using the manufacturers’ protocol. Real-Time PCR reactions (qRT-PCR) were performed using the high-throughput BioMark Real-Time PCR system (Fluidigm, South San Francisco, CA) and a 96-well plate. Further details have been described previously[19].

We normalized the raw cycle threshold (Ct) values for our main analysis using an adaptation of the global mean normalization method [20,21]. Methods like global mean normalization are useful and necessary when comparing expression levels between individuals, such as between individuals with and without cardiovascular disease. However, our candidate set of 61 CVD-related miRNA may not represent an appropriate sample of miRNA from which to calculate a global mean, therefore we additionally present results using raw Ct values as supplemental material. Standardized expression levels were calculated as the average expression of all miRNA in all participants in a sample (i.e. time 1) plus the difference between expression of a particular miRNA in that individual and sample and average expression of all miRNAs in that individual and sample. We added a standard global expression level back to global mean normalized values to transform all values back to positive values which is necessary for the coefficient of variation (CV) and intraclass correlation coefficient (ICC) calculations.

### Statistical Analysis

We imputed a Ct value of 28, the lower limit of detection for this particular assay, for samples with undetectable levels (higher values indicate fewer replicates and lower expression). A single copy of miRNA can be detected in 26–27 Ct in the BioMark System compared to 36–37 Ct in conventional qRT-PCR platforms. We calculated the CV for each miRNA as an average of each participant’s within-subject CV and considered CVs below 20% acceptable [22]. We calculated polychoric correlation coefficients instead of Spearman correlation coefficients to compare samples collected one year apart because this method is more appropriate for discretized data with multiple ties.

We also calculated the ICC to compare samples with 0, 24, and 48 hours delay to processing and samples collected one year apart. The ICC is the ratio of between-person variance to the total variance (between- and within-person variance) and takes into account absolute miRNA expression levels as measured by Ct value. We included the first within-person stability measurement (n = 80) in the delayed processing ICC calculation because the between-person variance was close to zero for most miRNAs among the young, healthy male volunteers. The combination of 80 older, less healthy HPFS participants and 12 young, healthy volunteers is more representative of a typical study population and generates more meaningful ICCs.

Between- and within-person variances were calculated using a mixed model where the participant was the random variable. We considered an ICC ≥ 0.4 adequate as lower values would greatly reduce our power to detect true and existing associations between miRNAs and disease in epidemiologic studies [8,22]. Furthermore, ICCs of 0.65 for serum cholesterol and 0.45 for plasma prolactin measured 2–3 years apart were associated with disease in previous studies [23,24].

### Ethics Statement

The study protocol was approved by the Institutional Review Board of the Brigham and Women's Hospital and by the Harvard T. H. Chan School of Public Health Human Subjects Committee Review Board and all participants provided written informed consent.

## Results

Expression levels ranged from 6.6 Ct to 28, where 28 represents no expression or zero copies of miRNA detected (Table 2). Many miRNAs were not detectable in all samples tested and most had low expression levels (Ct above 15).

### Inter-assay Reproducibility

Twenty-four of 61 miRNAs measured for the inter-assay reproducibility experiment had detectable expression levels in at least half of the study samples and 19 were detectable in ≥ 80% of samples (Table 3). Among these 24 miRNAs, 23 (96%) had average CVs of < 20%, but 14 of these 23 had a maximum CV ≥ 20% (of 15 pairs). Results were virtually identical using raw Ct values.

**Table 3:** Average and maximum pair CVs for standardized values of 61 miRNAs measured in 15 duplicate (split) samples.

Expressed at detectable levels in > 50% of samples					Expressed at detectable levels in ≤ 50% of samples				
Target meets established criteria	miRNA	Avg CV	Maximum CV	% Detected	Target meets established criteria	miRNA	Avg CV	Maximum CV	% Detected
Yes	hsa-miR-20b-5p	**2.6**	**7.3**	**50%**	Yes	hsa-miR-26b-3p	**2.4**	**6.2**	*3%*
Yes	hsa-miR-29b-3p	**3.8**	**8.8**	**100%**	Yes	hsa-miR-29a-3p	**2.2**	**6.2**	*3%*
Yes	hsa-miR-199a-3p	**3.5**	**9.9**	**87%**	Yes	hsa-miR-223-5p	**2.9**	**13.1**	*3%*
Yes	hsa-miR-26b-5p	**4.0**	**16.6**	**60%**	Yes	hsa-miR-145-5p	**2.0**	**6.2**	*10%*
Yes	hsa-miR-375	**5.7**	**14.9**	**53%**	Yes	hsa-miR-130b-3p	**2.5**	**7.9**	*7%*
Yes	hsa-miR-93-5p	**4.3**	**16.5**	**90%**	Yes	hsa-miR-140-3p	**3.0**	**7.2**	*33%*
Yes	hsa-miR-21-5p	**5.5**	**19.0**	**80%**	Yes	hsa-miR-222-3p	**2.2**	**6.6**	*40%*
Yes	hsa-miR-320a	**7.1**	**19.0**	**87%**	Yes	hsa-miR-122-5p	**4.1**	**15.3**	*13%*
Yes	hsa-miR-27b-3p	**8.0**	*40*.*1*	**100%**	Yes	hsa-miR-185-5p	**3.2**	**11.0**	*20%*
Yes	hsa-miR-17-5p	**6.6**	**19.9**	**67%**	Yes	hsa-let-7d-5p	**2.9**	**11.8**	*40%*
Yes	hsa-miR-30b-5p	**8.5**	*41*.*7*	**93%**	Yes	hsa-miR-126-5p	**4.8**	*22*.*0*	*10%*
Yes	hsa-miR-126-3p	**6.3**	*34*.*0*	**93%**	Yes	hsa-miR-133a	**3.4**	**10.1**	*27%*
Yes	hsa-miR-106b-5p	**7.6**	*30*.*7*	**93%**	Yes	hsa-miR-424-5p	**7.9**	*94*.*2*	*3%*
Yes	hsa-miR-130a-3p	**7.7**	*29*.*1*	**90%**	Yes	hsa-miR-23b-5p	**8.0**	*91*.*7*	*3%*
Yes	hsa-miR-27a-3p	**8.4**	*27*.*1*	**93%**	Yes	hsa-miR-424-3p	**8.3**	*94*.*2*	*3%*
Yes	hsa-miR-191-5p	**8.1**	*34*.*7*	**93%**	Yes	hsa-miR-155-5p	**9.8**	*80*.*0*	*7%*
Yes	hsa-miR-30c-5p	**9.3**	*40*.*1*	**100%**	No	hsa-miR-22-5p	23.7	*125*.*8*	*10%*
Yes	hsa-miR-26a-5p	**8.2**	*29*.*5*	**93%**	—	hsa-miR-151a-3p	NC	NC	*0%*
Yes	hsa-miR-221-3p	**9.3**	*32*.*8*	**93%**	—	hsa-miR-151a-5p	NC	NC	*0%*
Yes	hsa-miR-30e-3p	**13.9**	*45*.*9*	**100%**	—	hsa-miR-191-3p	NC	NC	*0%*
Yes	hsa-miR-409-3p	**11.6**	*31*.*7*	**70%**	—	hsa-miR-22-3p	NC	NC	*0%*
Yes	hsa-miR-320b	**18.6**	*83*.*1*	**100%**	—	hsa-miR-193b-3p	NC	NC	*0%*
Yes	hsa-miR-92a-3p	**11.9**	*62*.*2*	**93%**	—	hsa-miR-199a-5p	NC	NC	*0%*
No	hsa-miR-29c-3p	*20*.*7*	*58*.*1*	**100%**	—	hsa-let-7e-5p	NC	NC	*0%*
					—	hsa-miR-210	NC	NC	*0%*
					—	hsa-miR-361-5p	NC	NC	*0%*
					—	hsa-miR-142-5p	NC	NC	*0%*
					—	hsa-miR-23b-3p	NC	NC	*0%*
					—	hsa-miR-1	NC	NC	*0%*
					—	hsa-miR-25-3p	NC	NC	*0%*
					—	hsa-let-7d-3p	NC	NC	*0%*
					—	hsa-miR-10a-5p	NC	NC	*0%*
					—	hsa-miR-130b-5p	NC	NC	*0%*
					—	hsa-miR-133b	NC	NC	*0%*
					—	hsa-miR-145-3p	NC	NC	*0%*
					—	hsa-miR-29c-5p	NC	NC	*0%*
					—	hsa-miR-93-3p	NC	NC	*0%*

Bold indicates acceptable results and italics indicates unacceptable results. Avg CV = average CV; Max CV = maximum CV; % Detected = % samples with detectable levels; NC = not calculated.

### Delayed Processing Stability

**Fig 1:**
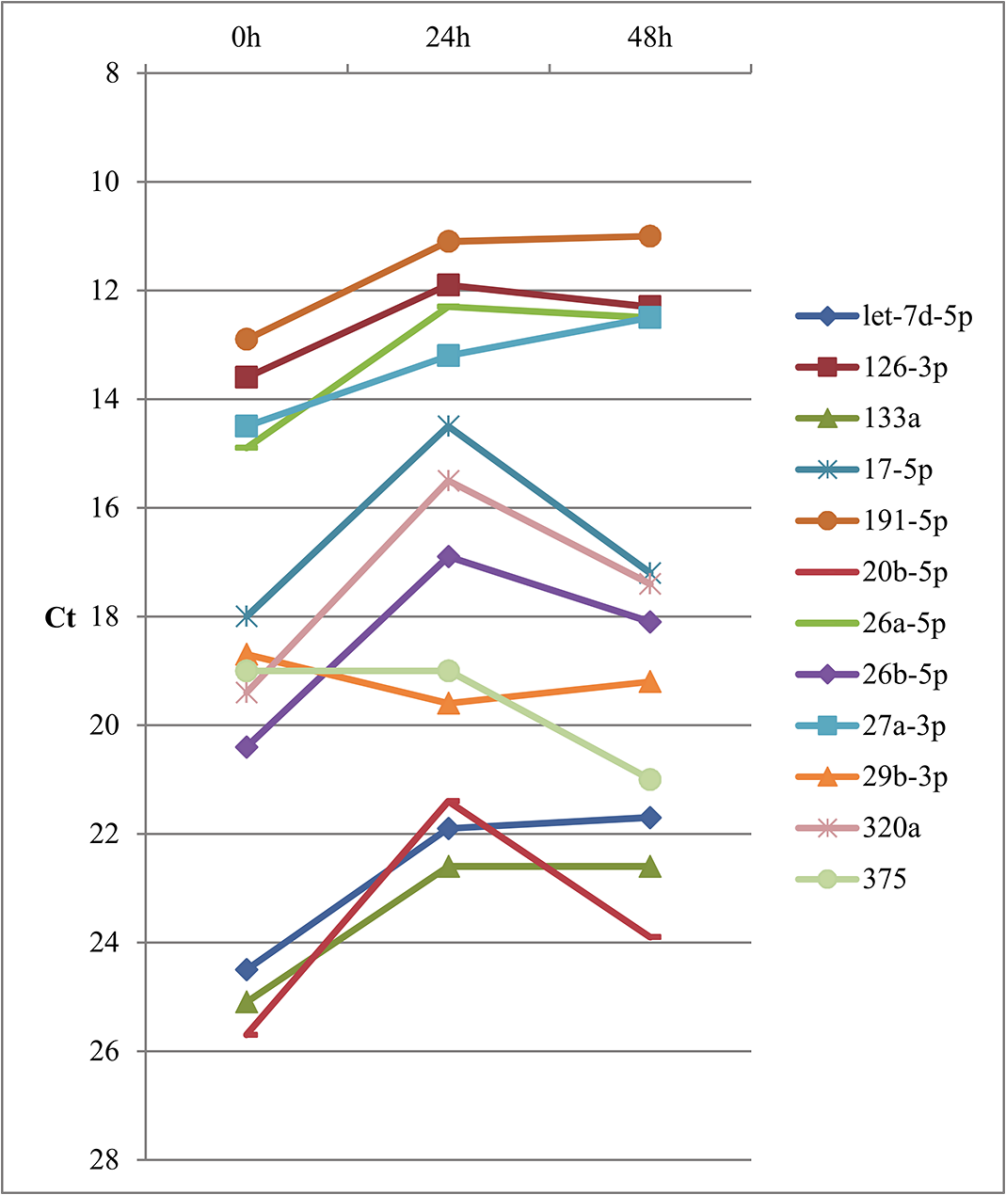
Expression levels of 12 miRNAs* with delayed processing of 0, 24, and 48 hours at 4°C. *12 miRNAs detectable in ≥ 50% of samples and with CVs below 20% for inter-assay reproducibility, delayed processing reproducibility, and short-term within-person stability, and ICCs ≥ 0.3 for short-term within-person stability.

Thirty-three of 61 miRNAs measured for the delayed processing experiment had detectable expression levels in at least half of the study samples and 29 were detectable in ≥ 80% of samples (S1 Table). Among these 33 miRNAs, 31 had average CVs of < 20%, but nine of these 31 had a maximum triplicate CV ≥ 20% (of 12 triplicates). Eleven of 61 miRNAs had ICCS > 0.4 across the different processing times (0, 24, and 48 hours at 4°C), indicating a high ratio of between-person variation to laboratory variability, but expression levels were low (above 15 Ct) for most miRNAs measured (Fig 1). Results were similar using raw Ct values (S2 Table).

### Within-Person Stability

Forty-one of 61 miRNAs measured for the within-person stability experiment had detectable expression levels in at least half of the study samples and 33 were detectable in ≥ 80% of samples (Table 4). Among these 41 miRNAs, 10 had average CVs of < 20% and an ICC above 0.40, but 6 of these 10 had a maximum CV ≥ 20% (of 80 pairs). Among these 10, polychoric correlation coefficients across 1–2 years ranged from 0.33 to 0.59. Using raw Ct values, 21 of 41 miRNAs had had an average CV of < 20% and an ICC above 0.40 (S3 Table).

**Table 4:** ICCs, average pair CVs, and correlation coefficients (r) using standardized values for 61 miRNAs measured in 80 participants one year apart.

Expressed at detectable levels in > 50% of samples							Expressed at detectable levels in ≤ 50% of samples						
Target meets established criteria	miRNA	ICC	Avg CV	Max CV	Polychoric r	% Detected	Target meets established criteria	miRNA	ICC	Avg CV	Max CV	Polychoric r	% Detected
Yes	hsa-miR-191-5p	**0.5**	**5.6**	*39*.*4*	0.59	**100%**	Yes	hsa-miR-145-3p	**0.6**	**2.4**	**10.7**	0.59	*2%*
Yes	hsa-miR-29c-3p	**0.5**	**8.2**	*29*.*7*	0.47	**100%**	Yes	hsa-miR-130b-5p	**0.6**	**2.4**	**10.7**	0.57	*1%*
Yes	hsa-miR-17-5p	**0.4**	**7.6**	*33*.*0*	0.45	**99%**	Yes	hsa-miR-199a-5p	**0.5**	**2.5**	**10.7**	0.53	*2%*
Yes	hsa-miR-133a	**0.4**	**6.4**	*29*.*3*	0.38	**59%**	Yes	hsa-miR-29c-5p	**0.5**	**2.6**	**10.7**	0.51	*3%*
Yes	hsa-let-7d-5p	**0.4**	**3.7**	**16.9**	0.39	**75%**	Yes	hsa-let-7d-3p	**0.5**	**2.6**	**12.4**	0.46	*1%*
Yes	hsa-miR-26a-5p	**0.4**	**7.2**	*21*.*6*	0.47	**99%**	Yes	hsa-miR-424-3p	**0.5**	**2.7**	*21*.*2*	0.54	*3%*
Yes	hsa-miR-375	**0.4**	**4.8**	**17.1**	0.47	**99%**	No	hsa-miR-191-3p	*0*.*3*	**2.9**	*27*.*5*	0.43	*5%*
Yes	hsa-miR-320a	**0.4**	**5.4**	**18.6**	0.38	**100%**	No	hsa-miR-10a-5p	*0*.*3*	**3.1**	**15.3**	0.36	*15%*
Yes	hsa-miR-130b-3p	**0.4**	**5.0**	**18.3**	0.33	**73%**	No	hsa-miR-26b-3p	*0*.*3*	**3.5**	**12.8**	0.33	*16%*
Yes	hsa-miR-27b-3p	**0.4**	**6.1**	*44*.*4*	0.46	**100%**	No	hsa-miR-133b	*0*.*3*	**3.0**	*34*.*8*	0.44	*4%*
No	hsa-miR-126-5p	*0*.*3*	**12.8**	*69*.*0*	0.36	**99%**	No	hsa-let-7e-5p	*0*.*2*	**3.7**	*23*.*3*	0.34	*14%*
No	hsa-miR-222-3p	*0*.*3*	**4.2**	*54*.*5*	0.48	**98%**	No	hsa-miR-22-3p	*0*.*2*	**3.6**	**19.5**	0.23	*24%*
No	hsa-miR-361-5p	*0*.*3*	**4.7**	**17.7**	0.31	**68%**	No	hsa-miR-142-5p	*0*.*2*	**3.2**	**10.9**	0.20	*28%*
No	hsa-miR-21-5p	*0*.*3*	**7.0**	*29*.*8*	0.35	**99%**	No	hsa-miR-151a-5p	*0*.*1*	**5.5**	*98*.*9*	0.27	*34%*
No	hsa-miR-126-3p	*0*.*3*	**4.3**	**19.8**	0.32	**100%**	No	hsa-miR-193b-3p	*0*.*1*	**5.8**	**17.5**	0.12	*48%*
No	hsa-miR-26b-5p	*0*.*3*	**7.3**	*21*.*8*	0.31	**97%**	**—**	hsa-miR-1	NC	NC	NC	NC	*0%*
No	hsa-miR-409-3p	*0*.*3*	**6.2**	*42*.*9*	0.62	**99%**	**—**	hsa-miR-155-5p	NC	NC	NC	NC	*0%*
No	hsa-miR-29b-3p	*0*.*3*	**6.8**	*28*.*2*	0.36	**100%**	**—**	hsa-miR-23b-3p	NC	NC	NC	NC	*0%*
No	hsa-miR-210	*0*.*3*	**8.6**	*24*.*9*	0.28	**84%**	**—**	hsa-miR-23b-5p	NC	NC	NC	NC	*0%*
No	hsa-miR-20b-5p	*0*.*3*	**4.8**	*23*.*3*	0.33	**100%**	**—**	hsa-miR-424-5p	NC	NC	NC	NC	*0%*
No	hsa-miR-140-3p	*0*.*2*	**4.7**	**14.8**	0.27	**91%**	** **						
No	hsa-miR-130a-3p	*0*.*2*	**4.3**	*29*.*5*	0.34	**99%**	** **						
No	hsa-miR-185-5p	*0*.*2*	**4.7**	**15.6**	0.20	**97%**	** **						
No	hsa-miR-223-5p	*0*.*2*	**6.8**	*122*.*1*	0.33	**96%**	** **						
No	hsa-miR-29a-3p	*0*.*2*	**4.5**	**16.7**	0.17	**98%**	** **						
No	hsa-miR-25-3p	*0*.*2*	**10.7**	*64*.*4*	0.18	**100%**	** **						
No	hsa-miR-145-5p	*0*.*2*	**4.9**	**14.7**	0.16	**76%**	** **						
No	hsa-miR-199a-3p	*0*.*2*	**4.0**	**13.2**	0.28	**100%**	** **						
No	hsa-miR-30c-5p	*0*.*2*	**7.3**	*47*.*2*	0.19	**100%**	** **						
No	hsa-miR-92a-3p	*0*.*2*	**9.2**	*37*.*9*	0.17	**100%**	** **						
No	hsa-miR-106b-5p	*0*.*1*	**5.5**	*25*.*8*	0.15	**100%**	** **						
No	hsa-miR-151a-3p	*0*.*1*	**5.6**	*36*.*5*	0.17	**78%**	** **						
No	hsa-miR-221-3p	*0*.*1*	**4.8**	*40*.*6*	0.22	**99%**	** **						
No	hsa-miR-93-5p	*0*.*1*	**5.5**	*20*.*4*	0.14	**100%**	** **						
No	hsa-miR-93-3p	*0*.*1*	**5.9**	*43*.*9*	0.15	**82%**	** **						
No	hsa-miR-122-5p	*0*.*1*	**7.2**	*24*.*8*	0.21	**51%**	** **						
No	hsa-miR-22-5p	*0*.*1*	**7.2**	*100*.*0*	0.17	**79%**	** **						
No	hsa-miR-30b-5p	*0*.*1*	**7.0**	*72*.*6*	0.15	**100%**	** **						
No	hsa-miR-27a-3p	*<0*.*1*	**5.4**	*23*.*5*	-0.03	**100%**	** **						
No	hsa-miR-320b	*<0*.*1*	**15.8**	*85*.*9*	0.38	**99%**	** **						
No	hsa-miR-30e-3p	*<0*.*1*	**17.3**	*114*.*2*	0.25	**99%**	** **						

Bold indicates acceptable results and italics indicates unacceptable results, NC = not calculated.

Table 5 summarizes our results for all three experiments. Almost all miRNAs that were expressed in more than 50% of samples had acceptable average CVs, although many of these had high maximum CVs. Although ICC’s with delayed processing were poor for several miRNAs, most studies do not have such delays and in these cases, inter-assay reproducibility and within-person stability over the short term are most relevant. Overall, six of 61 miRNAs satisfied criteria for inter-assay reproducibility and short-term within-person stability ICCs (miR-17-5p, -191-5p, -26a-5p, -27b-3p, -320a, and -375) however only two of these additionally met criteria for reproducibility with processing delays (miR-27b-3p and -26a-5p).

**Table 5:** Summary of reproducibility of 15 CVD-related miRNAs with CVs below 20% in duplicate samples, samples with processing delayed up to 48 hours, and samples collected 1–2 years apart.

	Split replicates		Delayed processing		Within-person 1–2 year stability		
miR	% Detected	Avg CV	% Detected	Avg CV	% Detected	Avg CV	ICC
let-7d-5p	40%	2.9	89%	7.7	75%	3.7	0.4
miR-126-3p	96%	6.3	100%	8.2	100%	4.3	0.3
miR-133a	27%	3.4	75%	7.6	59%	6.4	0.4
miR-17-5p	67%	6.6	100%	13.4	99%	7.6	0.4
miR-191-5p	93%	8.1	100%	11.1	100%	5.6	0.5
miR-20b-5p	50%	2.6	89%	9.5	100%	4.8	0.3
miR-26a-5p	93%	8.2	100%	12.2	99%	7.2	0.4
miR-26b-5p	60%	4.0	97%	11.3	97%	7.3	0.3
miR-27b-3p	100%	8.0	100%	7.2	100%	6.1	0.4
miR-29b-3p	100%	3.8	100%	6.8	100%	6.8	0.3
miR-320a	87%	7.1	100%	11.7	100%	5.4	0.4
miR-375	53%	5.7	94%	8.2	99%	4.8	0.4

## Discussion

Of 61 selected miRNAs, 24 (39%) were detected in at least 50% of inter-assay reproducibility samples, 33 (54%) in at least 50% of delayed processing samples, and 41 (67%) in at least 50% of within-person stability samples. Among those miRNAs detected in at least 50% of samples, 23 (96%) had average CVs below 20% for inter-assay reproducibility, 31 (94%) for delayed processing, and 41 (100%) for within-person stability over one year. Twelve miRNAs had acceptable CVs and were expressed in at least 50% of samples in all three experiments.

The 40 miRNAs with low within-person stability ICCs are not necessarily poorly measured, but may change acutely as a result of environmental or phenotypic changes. These rapidly changing miRNAs could be ideal markers of disease presence, severity, and subtype and may thus have substantial clinical relevance. However, for the purpose of risk prediction, these miRNAs are less stable and unlikely to predict risk of experiencing a cardiac event years in the future, especially with a single measurement. Although CVs were below 20% for most miRNAs, many of these miRNAs had high maximum CVs, where at least one individual had low reproducibility. Furthermore, although 31 miRNAs had acceptable CVs for the delayed processing experiment, several had low ICCs, indicating that when possible, temporary storage at 4°C before processing should be avoided.

Unlike the CV, the ICC takes within-assay variability into account relative to total variation. Therefore a higher CV may be acceptable if there is large between individual variation and a high ICC, but may not be acceptable if there is small between-individual variation and a low ICC, as in this particular case, because laboratory variability could be larger than between-person differences [7]. Our delayed processing study participants were similar by design, which limited between-individual variation: 11 of the 12 healthy men participating were between the ages of 19 and 33. ICCs and between-person variance were higher with the inclusion of the first within-person stability measurement (n = 80 less healthy, older men). Future studies with additional participants and greater between-individual variation may see more acceptable measures of variability.

Using standardized values for the short-term reproducibility study, total variation in miRNA expression was reduced and therefore ICCs were lower. However, standardizing with a small set of CVD-related miRNAs may not be an optimal normalization method. Our results suggest that using non-standardized candidates shows useful reproducibility, but further testing with truly global means may be necessary for adequate normalization.

It is difficult to calculate reproducibility for several miRNAs that were not detectable in most samples. If some miRNAs are only expressed under certain conditions, such as when disease is present, they may not have performed well in our experiments because the majority of our study participants were healthy. This does not necessarily mean they are poor biomarkers; if these miRNAs were only detectable when preclinical disease is present, they would represent ideal biomarkers for detection of asymptomatic disease. Nonetheless, where miRNAs may be studied specifically within healthy individuals, such as relating them to other biomarkers, our results suggest that a pilot testing phase is highly desirable to minimize futile studies.

Our study has several strengths, including the evaluation of inter-assay, delayed processing, and within-person stability over the short term using the gold-standard for gene expression, qRT-PCR. We focused our study on miRNAs that have been previously reported to be associated with varying types of cardiovascular disease and therefore represent interesting targets for future research. We further narrowed our miRNA target selection to candidates with established likelihood of detection in plasma (at least in some clinical scenarios), which are the most likely to be used in future epidemiological studies. Finally, to the best of our knowledge, ours is the first to explore reproducibility of miRNAs in long-term stored plasma samples.

Our study is not without limitation. We included healthy, male participants which limited between individual variation and miRNA expression levels in our study participants may not represent levels in less healthy men or in women. Furthermore, CVs may vary when using different laboratories and different study populations, such as those at a higher risk of developing chronic disease. Based on our experience, we would encourage investigators to evaluate laboratory, platform and sample-type specific assay performance prior to proceeding with large ventures.

In conclusion, six of 61 miRNAs selected met our criteria for acceptable inter-assay and short-term within-person reproducibility among those miRNAs with high expression levels. These miRNAs may represent targets for the future investigation of the associations between circulating miRNA expression and future CHD risk. Although these six miRNAs had acceptable CVs with delayed processing, ICCs were low for four indicating poor reproducibility without controlled temperature and time during processing. Stored plasma from large cohorts represents an exciting opportunity to further explore the biology of at least some miRNAs in the development of chronic diseases like CVD and cancer, however, many questions remain about the minimum and ideal requirements for such sample processing and storage to conduct valid analyses. To date, miRNA-disease relationships are not always reproducible across studies [25,26] or disease-specific [27], and sample collection and analysis techniques are not yet standardized [25].

## Supporting Information

S1 Table Average and maximum pair CVs and ICCs for standardized values of 61 miRNAs measured in 12 samples processed and frozen after 0, 24, and 48 hours at 4°C. Bold indicates acceptable results, italics indicates unacceptable results, and NC = not calculated. *Includes first within-person stability measurement (n = 80 HPFS participants).
(DOCX)

S2 Table Average and maximum pair CVs and ICCs for 61 miRNAs measured in 12 samples processed and frozen after 0, 24, and 48 hours at 4°C (raw Ct values). Bold indicates acceptable results, italics indicates unacceptable results, and NC = not calculated. *Includes first within-person stability measurement (n = 80 HPFS participants).
(DOCX)

S3 Table ICCs, average and maximum pair CVs, and correlation coefficients (r) for 61 miRNAs measured in 80 participants one year apart (raw Ct values). Bold indicates acceptable results, italics indicates unacceptable results, and NC = not calculated.
(DOCX)
