# Supplementary material for: Reproducibility of Circulating MicroRNAs in Stored Plasma Samples
Source: PLoS One. 2015 Aug 27;10(8):e0136665. doi: 10.1371/journal.pone.0136665 (PMC4552013; doi:10.1371/journal.pone.0136665)
Supplement: Supplementary file 1 [file pone.0136665.s001.docx]

Supplemental Table 1. Average and maximum pair CVs and ICCs for standardized values of 61 miRNAs measured in 12 samples processed and frozen after 0, 24, and 48 hours at 4°C.

Expressed at detectable levels in > 50% of samples						Expressed at detectable levels in ≤ 50% of samples					
Target meets established criteria	miRNA	Avg CV	Maximum CV	% Detected	ICC*	Target meets established criteria	miRNA	Avg CV	Maximum CV	% Detected	ICC*
Yes	hsa-miR-30c-5p	**5.8**	**11.5**	**100%**	**0.6**	Yes	hsa-miR-22-3p	**2.9**	**7.2**	*3%*	**0.5**
No	hsa-miR-140-3p	**6.4**	**9.9**	**81%**	*0.2*	Yes	hsa-miR-151a-3p	**3.0**	**7.2**	*3%*	**0.8**
No	hsa-miR-29b-3p	**6.8**	**11.9**	**100%**	*0.3*	No	hsa-miR-191-3p	**3.4**	**7.2**	*3%*	*<0.1*
No	hsa-miR-106b-5p	**6.9**	**12.0**	**100%**	*<0.1*	No	hsa-miR-142-5p	**3.5**	**6.7**	*31%*	*0.1*
Yes	hsa-miR-27b-3p	**7.2**	**15.6**	**100%**	**0.8**	No	hsa-miR-199a-5p	**4.1**	**11.6**	*11%*	*<0.1*
No	hsa-miR-185-5p	**7.2**	**12.8**	**92%**	*<0.1*	Yes	hsa-miR-361-5p	**4.1**	**9.4**	*17%*	**0.7**
No	hsa-miR-145-5p	**7.2**	**15.9**	**92%**	*0.1*	No	hsa-miR-193b-3p	**4.2**	**17.0**	*3%*	*0.2*
Yes	hsa-miR-133a	**7.6**	**17.1**	**75%**	**0.4**	Yes	hsa-miR-210	**4.3**	**12.7**	*14%*	**0.8**
No	hsa-let-7d-5p	**7.7**	**14.6**	**89%**	*0.2*	Yes	hsa-miR-223-5p	**4.3**	**9.5**	*22%*	**0.8**
No	hsa-miR-29a-3p	**8.1**	**14.3**	**67%**	*0.3*	No	hsa-let-7e-5p	**5.3**	**11.7**	*11%*	*<0.1*
No	hsa-miR-375	**8.2**	**18.1**	**94%**	*0.3*	No	hsa-miR-151a-5p	**6.2**	*40.6*	*3%*	*<0.1*
No	hsa-miR-126-3p	**8.2**	**12.1**	**100%**	*0.3*	No	hsa-miR-122-5p	**14.3**	*30.3*	*36%*	*<0.1*
No	hsa-miR-30b-5p	**8.3**	**14.5**	**100%**	*<0.1*	--	hsa-let-7d-3p	NC	NC	*0%*	NC
No	hsa-miR-27a-3p	**8.3**	**15.9**	**100%**	*<0.1*	--	hsa-miR-1	NC	NC	*0%*	NC
No	hsa-miR-93-5p	**8.5**	**13.5**	**100%**	*0.2*	--	hsa-miR-10a-5p	NC	NC	*0%*	NC
No	hsa-miR-199a-3p	**8.5**	*21.1*	**97%**	*<0.1*	--	hsa-miR-130b-5p	NC	NC	*0%*	NC
No	hsa-miR-222-3p	**8.6**	**15.6**	**92%**	*<0.1*	--	hsa-miR-133b	NC	NC	*0%*	NC
No	hsa-miR-221-3p	**8.7**	**16.9**	**100%**	*<0.1*	--	hsa-miR-145-3p	NC	NC	*0%*	NC
No	hsa-miR-130a-3p	**8.8**	**18.0**	**100%**	*<0.1*	--	hsa-miR-155-5p	NC	NC	*0%*	NC
Yes	hsa-miR-20b-5p	**9.5**	**15.1**	**89%**	**0.4**	--	hsa-miR-22-5p	NC	NC	*0%*	NC
No	hsa-miR-130b-3p	**10.7**	**16.7**	**58%**	*<0.1*	--	hsa-miR-23b-3p	NC	NC	*0%*	NC
No	hsa-miR-191-5p	**11.1**	**19.2**	**100%**	*0.2*	--	hsa-miR-23b-5p	NC	NC	*0%*	NC
No	hsa-miR-26b-5p	**11.3**	**18.1**	**97%**	*<0.1*	--	hsa-miR-25-3p	NC	NC	*0%*	NC
No	hsa-miR-320a	**11.7**	*20.5*	**100%**	*0.2*	--	hsa-miR-26b-3p	NC	NC	*0%*	NC
Yes	hsa-miR-92a-3p	**12.0**	*25.6*	**100%**	**0.4**	--	hsa-miR-29c-5p	NC	NC	*0%*	NC
No	hsa-miR-21-5p	**12.1**	*25.2*	**94%**	*<0.1*	--	hsa-miR-424-3p	NC	NC	*0%*	NC
Yes	hsa-miR-26a-5p	**12.2**	*25.3*	**100%**	**0.6**	--	hsa-miR-424-5p	NC	NC	*0%*	NC
No	hsa-miR-17-5p	**13.4**	*28.2*	**100%**	*<0.1*	--	hsa-miR-93-3p	NC	NC	*0%*	NC
No	hsa-miR-409-3p	**13.7**	*29.9*	**94%**	*0.3*						
No	hsa-miR-29c-3p	**14.0**	*31.3*	**100%**	*0.2*						
No	hsa-miR-30e-3p	**15.2**	*41.2*	**97%**	*0.3*						
No	hsa-miR-320b	*20.7*	*88.4*	**97%**	*<0.1*						
No	hsa-miR-126-5p	*22.9*	*41.2*	**78%**	*0.1*						

Bold indicates acceptable results, italics indicates unacceptable results, and NC = not calculated.

*Includes first within-person stability measurement (n=80 HPFS participants).
