# Supplementary material for: Reproducibility of Circulating MicroRNAs in Stored Plasma Samples
Source: PLoS One. 2015 Aug 27;10(8):e0136665. doi: 10.1371/journal.pone.0136665 (PMC4552013; doi:10.1371/journal.pone.0136665)
Supplement: Supplementary file 2 [file pone.0136665.s002.docx]

Supplemental Table 2. Average and maximum pair CVs and ICCs for 61 miRNAs measured in 12 samples processed and frozen after 0, 24, and 48 hou 4°C (raw Ct values).

**Expressed at detectable levels in > 50% of samples**						**Expressed at detectable levels in ≤ 50% of samples**				
**Target meets established criteria**	**miRNA**	**Avg CV**	**Maximum CV**	**% Detected**	**ICC***	**Target meets established criteria**	**miRNA**	**Avg CV**	**Maximum CV**	**% Detected**
Yes	hsa-miR-30c-5p	**8.3**	**15.5**	**100%**	**0.7**	No	hsa-miR-22-3p	**0.2**	**2.3**	*3%*
No	hsa-miR-140-3p	**7.2**	**12.8**	**81%**	*0.2*	Yes	hsa-miR-151a-3p	**0.4**	**4.3**	*3%*
Yes	hsa-miR-29b-3p	**4.4**	**8.1**	**100%**	**0.7**	No	hsa-miR-191-3p	**0.5**	**6.0**	*3%*
No	hsa-miR-106b-5p	**8.9**	**16.1**	**100%**	*0.3*	No	hsa-miR-142-5p	**3.5**	**10.3**	*31%*
Yes	hsa-miR-27b-3p	**7.7**	**13.4**	**100%**	**0.8**	No	hsa-miR-199a-5p	**2.5**	**13.6**	*11%*
No	hsa-miR-185-5p	**9.1**	**17.7**	**92%**	*<0.1*	Yes	hsa-miR-361-5p	**3.0**	**12.3**	*17%*
No	hsa-miR-145-5p	**9.2**	**18.7**	**92%**	*0.2*	Yes	hsa-miR-193b-3p	**1.3**	**15.3**	*3%*
Yes	hsa-miR-133a	**9.8**	**17.0**	**75%**	**0.4**	Yes	hsa-miR-210	**2.4**	**13.5**	*14%*
No	hsa-let-7d-5p	**10.0**	**17.9**	**89%**	*0.2*	Yes	hsa-miR-223-5p	**3.7**	**12.5**	*22%*
No	hsa-miR-29a-3p	**9.2**	**17.6**	**67%**	*0.3*	No	hsa-let-7e-5p	**3.3**	**13.0**	*11%*
No	hsa-miR-375	**8.6**	*26.8*	**94%**	*0.2*	No	hsa-miR-151a-5p	**3.6**	*42.8*	*3%*
No	hsa-miR-126-3p	**11.6**	*20.5*	**100%**	*0.2*	No	hsa-miR-122-5p	**12.7**	*27.6*	*36%*
No	hsa-miR-30b-5p	**10.3**	**19.6**	**100%**	*<0.1*	--	hsa-let-7d-3p	NC	NC	*0%*
No	hsa-miR-27a-3p	**11.2**	*21.9*	**100%**	*0.2*	--	hsa-miR-1	NC	NC	*0%*
No	hsa-miR-93-5p	**9.4**	**17.5**	**100%**	*0.3*	--	hsa-miR-10a-5p	NC	NC	*0%*
No	hsa-miR-199a-3p	**11.3**	*25.4*	**97%**	*<0.1*	--	hsa-miR-130b-5p	NC	NC	*0%*
No	hsa-miR-222-3p	**10.8**	**18.2**	**92%**	*<0.1*	--	hsa-miR-133b	NC	NC	*0%*
No	hsa-miR-221-3p	**12.6**	*23.0*	**100%**	*0.1*	--	hsa-miR-145-3p	NC	NC	*0%*
No	hsa-miR-130a-3p	**11.6**	*23.2*	**100%**	*<0.1*	--	hsa-miR-155-5p	NC	NC	*0%*
Yes	hsa-miR-20b-5p	**10.3**	**18.4**	**89%**	**0.4**	--	hsa-miR-22-5p	NC	NC	*0%*
No	hsa-miR-130b-3p	**12.0**	*20.1*	**58%**	*<0.1*	--	hsa-miR-23b-3p	NC	NC	*0%*
No	hsa-miR-191-5p	**14.0**	*26.3*	**100%**	*0.3*	--	hsa-miR-23b-5p	NC	NC	*0%*
No	hsa-miR-26b-5p	**13.2**	*24.6*	**97%**	*0.1*	--	hsa-miR-25-3p	NC	NC	*0%*
No	hsa-miR-320a	**13.2**	*22.9*	**100%**	*0.2*	--	hsa-miR-26b-3p	NC	NC	*0%*
Yes	hsa-miR-92a-3p	**10.8**	**18.7**	**100%**	**0.6**	--	hsa-miR-29c-5p	NC	NC	*0%*
No	hsa-miR-21-5p	**14.4**	*29.2*	**94%**	*<0.1*	--	hsa-miR-424-3p	NC	NC	*0%*
Yes	hsa-miR-26a-5p	**14.9**	*31.0*	**100%**	**0.6**	--	hsa-miR-424-5p	NC	NC	*0%*
No	hsa-miR-17-5p	**15.6**	*37.3*	**100%**	*<0.1*	--	hsa-miR-93-3p	NC	NC	*0%*
No	hsa-miR-409-3p	**16.1**	*33.8*	**94%**	*0.2*					
No	hsa-miR-29c-3p	**16.0**	*33.0*	**100%**	*0.3*					
No	hsa-miR-30e-3p	**16.0**	*44.9*	**97%**	*0.3*					
No	hsa-miR-320b	**15.8**	*90.4*	**97%**	*<0.1*					
No	hsa-miR-126-5p	*24.2*	*45.8*	**78%**	*0.1*					

Bold indicates acceptable results, italics indicates unacceptable results, and NC = not calculated.
* Includes first within-person stability measurement (n=80 HPFS participants).
