# Supplementary material for: Reproducibility of Circulating MicroRNAs in Stored Plasma Samples
Source: PLoS One. 2015 Aug 27;10(8):e0136665. doi: 10.1371/journal.pone.0136665 (PMC4552013; doi:10.1371/journal.pone.0136665)
Supplement: Supplementary file 3 [file pone.0136665.s003.docx]

Supplemental Table 3. ICCs, average and maximum pair CVs, and correlation coefficients (r) for 61 miRNAs measured in 80 participants one year apart (raw Ct values).

Expressed at detectable levels in > 50% of samples						
**Target meets established criteria**	**miRNA**	**ICC**	**Avg CV**	**Max CV**	**Polychoric r**	**% Detected**
Yes	hsa-miR-191-5p	**0.7**	**7.9**	*36.8*	0.64	**100%**
Yes	hsa-miR-29c-3p	**0.6**	**10.5**	*36.1*	0.58	**100%**
Yes	hsa-miR-17-5p	**0.5**	**10.3**	*41.1*	0.55	**99%**
Yes	hsa-miR-133a	**0.5**	**7.4**	*35.7*	0.58	**59%**
Yes	hsa-let-7d-5p	**0.5**	**5.5**	*25.1*	0.49	**75%**
Yes	hsa-miR-26a-5p	**0.5**	**9.7**	*30.8*	0.51	**99%**
No	hsa-miR-375	*0.3*	**5.0**	*26.9*	0.46	**99%**
Yes	hsa-miR-320a	**0.6**	**6.4**	*26.3*	0.63	**100%**
Yes	hsa-miR-130b-3p	**0.6**	**5.9**	**18.1**	0.60	**73%**
Yes	hsa-miR-27b-3p	**0.7**	**6.7**	*43.7*	0.63	**100%**
No	hsa-miR-126-5p	*0.2*	**14.6**	*79.3*	0.29	**99%**
Yes	hsa-miR-222-3p	**0.5**	**5.9**	*33.8*	0.57	**98%**
Yes	hsa-miR-361-5p	**0.5**	**5.9**	*27.6*	0.49	**68%**
No	hsa-miR-21-5p	*0.4*	**9.9**	*41.0*	0.41	**99%**
Yes	hsa-miR-126-3p	**0.6**	**7.0**	*21.1*	0.60	**100%**
Yes	hsa-miR-26b-5p	**0.4**	**10.5**	*34.7*	0.45	**97%**
No	hsa-miR-409-3p	*0.1*	**8.1**	*45.9*	0.30	**99%**
Yes	hsa-miR-29b-3p	**0.7**	**4.5**	*26.5*	0.64	**100%**
Yes	hsa-miR-210	**0.4**	**9.7**	*30.1*	0.44	**84%**
Yes	hsa-miR-20b-5p	**0.5**	**6.0**	*30.0*	0.50	**100%**
Yes	hsa-miR-140-3p	**0.5**	**5.6**	**19.7**	0.49	**91%**
No	hsa-miR-130a-3p	*0.3*	**6.8**	*40.6*	0.43	**99%**
No	hsa-miR-185-5p	*0.4*	**6.0**	*22.1*	0.40	**97%**
No	hsa-miR-223-5p	*0.2*	**8.5**	*102.3*	0.22	**96%**
No	hsa-miR-29a-3p	*0.4*	**5.9**	*20.0*	0.37	**98%**
Yes	hsa-miR-25-3p	**0.5**	**11.1**	*53.0*	0.54	**100%**
No	hsa-miR-145-5p	*0.4*	**6.2**	*23.2*	0.39	**76%**
Yes	hsa-miR-199a-3p	**0.4**	**6.3**	**15.8**	0.43	**100%**
Yes	hsa-miR-30c-5p	**0.4**	**10.1**	*56.9*	0.44	**100%**
No	hsa-miR-92a-3p	*0.3*	**9.1**	*59.4*	0.50	**100%**
No	hsa-miR-106b-5p	*0.4*	**8.4**	*40.6*	0.46	**100%**
No	hsa-miR-151a-3p	*0.1*	**7.9**	*40.6*	0.07	**78%**
No	hsa-miR-221-3p	*0.3*	**7.7**	*51.6*	0.42	**99%**
Yes	hsa-miR-93-5p	**0.4**	**6.9**	*31.3*	0.49	**100%**
No	hsa-miR-93-3p	*0.1*	**7.5**	*43.5*	0.15	**82%**
No	hsa-miR-122-5p	*0.4*	**7.1**	*26.0*	0.39	**51%**
No	hsa-miR-22-5p	*<0.1*	**9.2**	*98.6*	-0.01	**79%**
No	hsa-miR-30b-5p	*0.3*	**11.0**	*75.4*	0.41	**100%**
No	hsa-miR-27a-3p	*0.3*	**8.1**	*36.5*	0.30	**100%**
No	hsa-miR-320b	*<0.1*	**10.0**	*86.5*	-0.09	**99%**
No	hsa-miR-30e-3p	*<0.1*	**15.8**	*109.9*	0.02	**99%**

Expressed at detectable levels in ≤ 50% of samples					
**Target meets established criteria**	**miRNA**	**ICC**	**Avg CV**	**Max CV**	**Polychoric r**
No	hsa-miR-145-3p	*<0.1*	**0.1**	**3.6**	NC
No	hsa-miR-130b-5p	*<0.1*	**0.2**	**11.3**	NC
No	hsa-miR-199a-5p	*<0.1*	**0.2**	**5.9**	NC
No	hsa-miR-29c-5p	*<0.1*	**0.4**	**10.0**	NC
No	hsa-let-7d-3p	*<0.1*	**0.2**	**10.3**	NC
No	hsa-miR-424-3p	*<0.1*	**0.5**	**19.0**	NC
No	hsa-miR-191-3p	*<0.1*	**1.1**	*26.1*	NC
No	hsa-miR-10a-5p	*0.3*	**1.4**	*20.1*	0.52
No	hsa-miR-26b-3p	*<0.1*	**2.3**	*21.2*	NC
No	hsa-miR-133b	*0.1*	**1.0**	*43.1*	0.58
No	hsa-let-7e-5p	*0.1*	**2.6**	*27.9*	0.36
No	hsa-miR-22-3p	*0.2*	**3.1**	*23.7*	0.29
No	hsa-miR-142-5p	*0.4*	**2.6**	**15.6**	0.45
No	hsa-miR-151a-5p	*0.2*	**5.4**	*104.4*	0.21
Yes	hsa-miR-193b-3p	**0.4**	**5.0**	**18.8**	0.49
--	hsa-miR-1	NC	NC	NC	NC
--	hsa-miR-155-5p	NC	NC	NC	NC
--	hsa-miR-23b-3p	NC	NC	NC	NC
--	hsa-miR-23b-5p	NC	NC	NC	NC
--	hsa-miR-424-5p	NC	NC	NC	NC

Bold indicates acceptable results, italics indicates unacceptable results, and NC = not calculated.
